# The Application of Stimuli-Sensitive Actuators Based on Graphene Materials

**DOI:** 10.3389/fchem.2019.00803

**Published:** 2019-12-10

**Authors:** Jiangli Xue, Zhaoshun Gao, Liye Xiao

**Affiliations:** Interdisciplinary Research Center, Institute of Electrical Engineering, Chinese Academy of Science, Beijing, China

**Keywords:** graphene, actuators, stimulation, electrical, electrochemical, humidity, light, thermal

## Abstract

Graphene-based materials that can spontaneously response to external stimulations have triggered rapidly increasing research interest for developing smart devices due to their excellent electrical, mechanical and thermal properties. The specific behaviors as bending, curling, and swing are benefit for designing and fabricating the smart actuation system. In this minireview, we overview and summarize some of the recent advancements of stimuli-responsive actuators based on graphene materials. The external stimulus usually is as electrical, electrochemical, humid, photonic, and thermal. The advancement and industrialization of graphene preparation technology would push forward the rapid progress of graphene-based actuators and broaden their application including smart sensors, robots, artificial muscles, intelligent switch, and so on.

## Introduction

Artificial actuators are stimuli-sensitive devices that can undergo a reversible change in shape, volume, modulus, or some other mechanical properties in response to various external stimuli, which have attracted tremendous attentions attribute to the wide applications for robots, intelligent switch, smart sensors, memory chips, and prosthetic devices (Kim and Lieber, 1999; Jang et al., 2008; Huang et al., 2012; Xue et al., 2015; Xu et al., 2018b). Usually, the external stimuli can be electrical, optical, thermal, humid, and other forms of stimulation depending on the actuation mechanism (Huang et al., 2012). The traditional materials using for actuators generally have their own limitations. For example, piezoelectric or ferroelectric ceramics show low mechanical flexibility leading to slow response, and conducting polymer materials have suffered from high driving voltages and low energy conversion efficiency. Therefore, developing the advanced materials or the compatible fabrication methods for large displacement and a rapid response have been the topic of research during the past decade. To date, carbon-based nanomaterials have been caused great concern for proposing as appropriate candidates for actuators due to the large deformation either in planar or spatial warping (Baughman et al., 1999; Deng et al., 2017). Recently, *Peng* group reported the mechanically actuating fibers by carbon nanotube displaying a rapid response for using as the smart window and louvers (He et al., 2015).

Graphene, as another carbon nanomaterial with just one atom layer thick, that features high electron conductivity, excellent mechanical flexibility, huge surface area, superior thermal conductivity and stability, has captured a broad research interest for using as material or structural component in the actuator fabrication (Novoselov et al., 2004; Zan et al., 2011; Cheng et al., 2016; Xu et al., 2018b; Liu Y. Q. et al., 2019). It could be not only served as the electrode in the electrically/electrochemically driven actuators, but also the energy converting component or active actuation component in the photothermal systems, as well as in the moisture actuators (Yang et al., 2018). For example, the composite graphene fiber can be elongation or contraction under electrochemical stimulus (Wang Y. H. et al., 2013) or serve as a rotational motor under moisture-driven situations (Cheng et al., 2014). As we all know, great progress has been made in studying of actuation system based on graphene materials in only few years. Therefore, to organize an article on graphene-based actuator for quick understanding the limitation and strengths is highly demanded.

**Figure 1:**
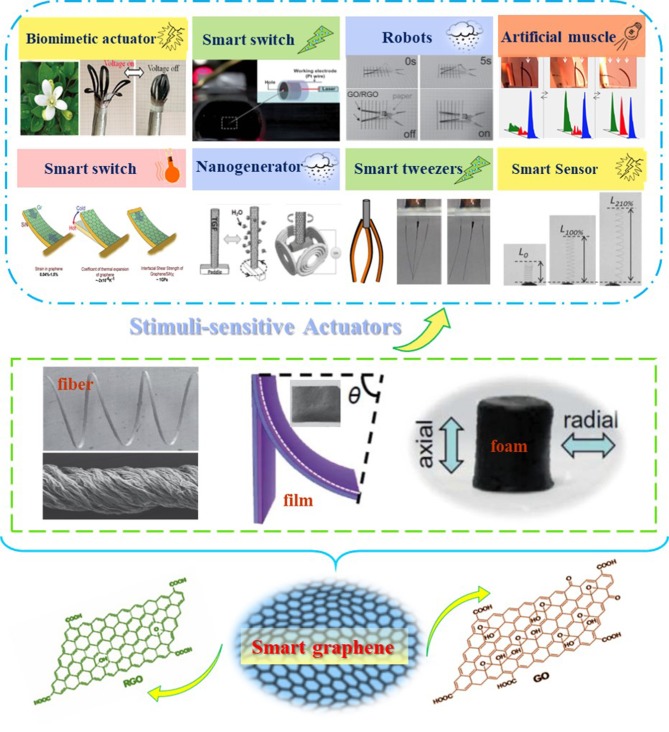
Stimuli-responsive graphene systems toward actuator applications. Reproduced from Novoselov et al. (2004) with permission of Science. Reproduced from Hummers and Offeman (1958) with permission of American Chemical Society. Reproduced from Cheng et al. (2014) with permission of Royal Society of Chemistry. Reproduced from Cheng et al. (2014) with permission of WILEY-VCH. Reproduced from Liu et al. (2012a) with permission of Royal Society of Chemistry. Reproduced from Liu et al. (2012b) with permission of Royal Society of Chemistry. Reproduced from Chang et al. (2019) with permission of WILEY-VCH. Reproduced from Han et al. (2015) with permission of WILEY-VCH. Reproduced from Cheng et al. (2016) with permission of American Chemical Society. Reproduced from Conley et al. (2011) with permission of American Chemical Society. Reproduced from Wang Y. H. et al. (2013) with permission of Elsevier.

In the minireview, we summarize the recent developments of graphene-based actuation systems and the discuss the application of graphene-based actuators under various external stimuli like electrical, electrochemical, humid, light, and other stimulations (Figure 1).

## The Application of Actuators Under Different Stimuli

Generally, the graphene-based stimuli-responsive actuators are the unique devices that can convert an external stimulus into automatic action. For the sample of isotropy, it shows planar expansion or contraction (Liu et al., 2012b; Hu et al., 2014; Xue et al., 2015), and for the sample with the construction of asymmetric, it shows spatial warping (Liu et al., 2012a; Wang Y. H. et al., 2013). The deformation of graphene is mainly caused by the stretch or rotation of carbon-carbon bands, and the hydrophilic and hydrophobicity of graphene-based materials is another reason inducing the deformation (Xu et al., 2018b). Recently, a novel asymmetric unimorph actuator based on graphene hydrogel was reported exhibiting synchronous responses for the stimulus of both photonic and humidifying/dehumidifying (Yang et al., 2019). In this section, we will briefly summarize the graphene-based actuator induced by some typical external stimuli like electrical, electrochemical, humid, light, and heat.

## Electrically Stimulated Actuation

The electrical-stimulus actuators are the most widely research transferring electrical energy/signal to mechanical energy/change (Zhao et al., 2013). Since the related work using graphene as resonator was firstly reported by *McEuen* group that showing the highest modulus than any other materials (Sazonova et al., 2004; Bunch et al., 2007), graphene materials based electrical stimulus-sensitive actuators have attracted a great deal of attention. Zhu et al. fabricated a bimorph actuator based on graphene materials with a large displacement and rapid response under low power consumption (Zhu et al., 2011). Later on, *Jang* group reported an acoustic actuator using a transparent and flexible graphene film as electrode which shown higher responses over all frequencies than those using a commercial PEDOT:PPS film as electrode (Shin et al., 2011). It could be used as loudspeaker with the properties of thin and lightweight.

By using the pure graphene with isotropy, *Chen* group reported a spongy graphene paper with an inside foldable corrugated structure exhibited reversible macroscopical length contraction with a strain of 2.4% under 10 V (Hu et al., 2014). Although the deformation efficiency is large, the power consumption is too much. Soon afterwards, our group put forward a strategy for the controllable fabrication of re-shaped graphene hydrogel showing the large strain up to 2.9% under very low voltage of 0.8 V (Xue et al., 2015).

A very recent and interesting study was reported by *Wang* group (Chang et al., 2019). The soft actuator with the construction of asymmetric based on graphene/polypropylene was fabricated by adhesion the polypropylene film on one side of the drop-coating graphene film. This actuator exhibited large, fast, and reversible deformation in response to electrical stimuli due to their inherent quality for high electrothermal and thermal expansion ability. Furthermore, the biomimetic devices like artificial flowers could be construct by using this actuator, which opens the way for constructing smart devices in the practical biomimetic applications.

## Electrochemically Stimulated Actuation

Electrochemically stimulated actuator is another widely researched device involving the actuation caused by ion-doping. Unimorph actuator with a single layer architecture have been demonstrated previously (Sansiñena et al., 2003; Han and Shi, 2006; Baker et al., 2008). Xie et al. designed and prepared a unique unimorph actuator based on a monolithic graphene film with asymmetrically modified surfaces by treating with O_2_ and hexane plasma on different sides of graphene film (Xie et al., 2010). Even so, most unimorph actuators consist of a bilayer or multilayer. For example, Liang et al. demonstrated an electrochemical actuator constructed on free-standing graphene/Fe_3_O_4_ hybrid papers providing increased actuation strain over the pristine graphene papers (Liang et al., 2011).

*Qu* group later reported that a kind of actuators made of asymmetrically graphene/polypyrrole films (Liu et al., 2012a) and fibers (Wang Y. H. et al., 2013), which not only exhibiting a large bending angle movement but also for use in multi-armed tweezers. In addition, they also constructed the 3D uniform network architecture of graphene/polypyrrole actuator with a record strain of 2.5% (Liu et al., 2012b), even up to 13.5% (Xue et al., 2015) after re-shaping strategy, under a square wave potential of 0.8 V. These actuators based on the 3D structure can be used as smart switch and even provide immense potential for the design of new types of actuators.

## Humidly Stimulated Actuation

As we all know, graphene is a kind of hydrophobic material which has been successfully prepared by mechanical exfoliation (Novoselov et al., 2004), chemical vapor deposition on metal (Cu and Ni) substrates (Hao et al., 2013; Lin et al., 2019; Liu C. et al., 2019), epitaxial growth (Yang et al., 2013), and so on. Whereas, graphene oxide as a derivative of graphene is derives from Hummers' method with numerous oxygen-related functional groups inducing fast adsorption and desorption of water molecules (Hummers and Offeman, 1958; Zhu et al., 2012; Han et al., 2017). Therefore, another kind of actuators based on the hydrophilic and hydrophobic of graphene materials could be construct. The deformation is mainly related to the change of water amounts or relative humidity in the environment (Zhao et al., 2013).

Guo et al. investigated GO layers fold and unfold behavior under a humidity changed environment, which opens up a possibility for GO as smart stimuli-response materials (Guo et al., 2011). Later on, Cheng et al. designed a region-asymmetric graphene/graphene oxide fiber actuator via laser reduction of fresh spun graphene oxide fibers (Cheng et al., 2013). By controlling the reduction region-specifically of graphene oxide, the complex deformation of this fiber actuator had been established when the relative humidity changed. Meanwhile, the crawler robot was made which could walk unidirectionally in a step-by-step manner with periodical alternation of the humidity in the environment. In another study, the twist graphene fiber fabricated by rotary of GO fiber can serve as a rotational motor under moisture-driven (Cheng et al., 2014). Before long, the asymmetrical reduced graphene oxide/graphene oxide bilayer structure was constructed by focusing sunlight irradiation on a graphene oxide paper (Han et al., 2015). The similar crawler robot but with the “leg” and “body” was also established that can move forward when the relative humidity change alternatively. Therefore, the moisture-sensitive actuators based on graphene/graphene oxide materials are the potential for developing the application of soft robot.

In recent years, another kind of moisture-sensitive devices maybe called power nanogenerators have attracted much attention through the mechanism is still debatable (Xu et al., 2018b). *Qu* group reported a lot of related and nice works (Zhao et al., 2015, 2016a, 2017; Liang et al., 2017, 2018; Xu et al., 2018a). For example, Zhao et al. provided electric generators for the first time by using the graphene oxide film with the oxygen-group gradient to harvest energy from moisture environment (Zhao et al., 2015).

## Light Stimuli Actuation

Graphene materials could convert the energy of light to heat or mechanical energy and display excellent photo-responsive (Acik et al., 2010; Robinson et al., 2011). An optically driven actuators based on graphene materials can be used as a device with possibilities of wireless actuation, remote displacement controls and robotic motions (Zhao et al., 2013). Wang et al. created near-infrared light-driven actuators based on reduced graphene sheet exhibiting rapid and tunable bending motions at the specific position controlled by light inducing finger-like flexing and crawling (Wang E. et al., 2013). More recent, *Liu* group designed a bilayer actuator combining the two materials of graphene and PDMS showing the highest deformation. This device was benefit to construct the beluga whale soft robot through improving the photo-responsive deflection and deflecting the speed of these actuators (Wang et al., 2019). From the above, the photo-sensitive actuators based on graphene materials have high deformation and controllable site causing a wide variety application including artificial muscles and soft robots.

## Thermally Stimulated Actuation

Theoretically, graphene has demonstrated distinctively large negative coefficient of thermal expansion (CTE). The value is −7 ppm K^−1^ in a single-layer (Bao et al., 2009) and −81.2 ppm K^−1^ in a discotic liquid crystal phase (Grigoriadis et al., 2010; Zhao et al., 2016b). Conley et al. developed a bilayer actuator constructed by graphene materials attached on different metal substrate like SiN_x_ and Au in order to probe the interaction between graphene and other metal. It was shown that all the cantilevers were bent toward the graphene side at room temperature and the bending degree related to the gradient of temperature, yet the strain was larger based on graphene/SiNx than graphene/Au (Conley et al., 2011). Kim et al. demonstrated a simple method to fabricate graphene oxide/Nylon-6,6 polymer composite fibers which could lift loads over 100 times heavier than itself and exhibit multi-directional actuation with the change of temperature (Kim et al., 2018). Therefore, the novel switch and actuator would be created by using graphene materials under thermal stimuli.

## Conclusion and Outlook

In summary, the recent researches on graphene materials for the devices of actuators have brought up a great deal of government and social attention and support. Particularly, stimuli-sensitive actuators based on graphene materials which spontaneously respond to external stimuli will be given rise to a promising field for developing novel smart devices. The great progress has been made for stimuli-responsive actuation systems that could convert electrical, chemical, photonic, thermal and other types energy/signal to mechanical energy/action. Yet, there are several challenges to overcome like the problem of the graphene production, the unsatisfactory mechanical strength of graphene and the effective of actuation response and so on. Therefore, development of advanced technique and promotion of industrialization are demanded to realize the variety practical applications.

## Author Contributions

All authors listed have made a substantial, direct and intellectual contribution to the work, and approved it for publication.

### Conflict of Interest

The authors declare that the research was conducted in the absence of any commercial or financial relationships that could be construed as a potential conflict of interest.
